# Estimations of child linguistic productivity controlling for vocabulary and sample size

**DOI:** 10.3389/fpsyg.2022.996610

**Published:** 2022-10-18

**Authors:** Javier Aguado-Orea

**Affiliations:** Centre for Behavioural Science and Applied Psychology (CeBSAP), Sheffield Hallam University, Sheffield, United Kingdom

**Keywords:** linguistic productivity, verb inflection, subject agreement, acquisition of English, acquisition of Spanish, cognitive development

## Abstract

Children’s use of present tense suffixes is less productive than that of their parents, after correcting for sample size and lexical knowledge, according to a recently established approach for the study of inflectional productivity. This article expands on this technique by providing precise estimates of early grammatical productivity through systematic random sampling and allowing for developmental assessment. Two cross-linguistic comparisons are given in the results of this study. Two Spanish-speaking children and their parents are compared with four English-speaking children and their parents. The second comparison examines potential differences in productivity throughout developmental stages using the same six children’s speech. The findings indicate that Spanish-acquiring children are less productive than their parents while utilising the paradigm under study, but that productivity levels increase over time. In contrast, the English-speaking children’s morphosyntactic production mirrors that of their parents. Although the primary focus of this research is methodological, these findings have consequences for theoretical theories arguing either rule abstraction or a restricted generalisation of early exemplars.

## Introduction

The primary objective of this article is to describe and disseminate a novel method for estimating changes in the productive use of grammatical knowledge over time. In the first section of this introduction, the significance of functional knowledge analyses in the study of cognitive development is explained briefly. It also discusses potential problems associated with these types of analyses and how solutions can be provided. This section is followed by a more in-depth examination of the theoretical implications of discovering differences in the productive use of grammatical knowledge, with reference to a classic debate between rule-based and exemplar-based knowledge. The present technique is described in greater depth in the third section.

### Challenges and solutions for the assessment of productive knowledge

Around their second year of life, children begin combining words into sentences. For example, children learning English as a first language must learn that the pronoun ‘I’ must be used to refer to themselves when expressing an action (e.g., ‘I want it’). Previous research has sought to answer the question of whether these types of expressions are constructed through the application of rule-based knowledge or whether they are initially more formulaic. If children are already applying rules, they should have mastered the skills necessary to perform combinatorial operations involving verbs and pronouns. If they are still employing less abstract knowledge, they may be using unanalysed expressions in particular contexts. For example, because ‘I-want-it’ was effective when other people were attempting to obtain something, children may use this expression without fully analysing its components. In other words, although the sentence contains a subject pronoun, this grammatical value has not yet been attained for the speaker. The method presented here aims to demonstrate a technique for estimating the cognitive properties of early language constructions by measuring the productive use of particular lexical items. Returning to the same example, it involves determining the extent to which ‘I’ can be considered to be used with multiple verbs.

It is important to note that, despite the fact that the present study focuses on grammatical knowledge, its significance permeates the entire field of cognitive development. It has been traditionally assumed that very young children can only make representations of their immediate sensory world, and that they gradually acquire a more symbolic understanding (Piaget and Inhelder, 2000). Thus, although children begin to comprehend words at a young age, they do so in a limited capacity. Around the sixth month of life, first words refer to only specific objects (e.g., “mummy”), and then, around the first year of life, they begin to represent categories of objects (e.g., “dog”) (Campbell and Hall, 2022). This gradual increase in abstract knowledge occurs across a vast array of cognitive domains, such as numerical knowledge (e.g., Spelke, 2022). However, although the concept of identifying intermediate stages of complexity is conceptually alluring, in practice it is a difficult task because knowledge must be estimated from behavioural responses. As a result, authors have concentrated on specific cognitive domains (e.g., visual perception, causality, and numerical understanding) and have attempted to define a paradigm that can be used to measure different levels of productive knowledge. Therefore, any study interested in observing changes in abstractness must first choose a well-defined set of possible processing operations (Karmiloff-Smith, 1992). The purpose of the following paragraphs is to describe the chosen research paradigm.

Previous research has examined the productive use of determiners in English, taking into account the proportion of nouns paired with “a/an” and “the” in samples of spontaneous speech (Pine et al., 2013). Children use nouns in a more restricted manner (i.e., with fewer determiners) than their caregivers. Other studies have looked into the productive use of verbs with various affixes. In English, the variation would be between one and three units, given that verbs contain three possible morphemes (e.g., “plays,” “played” and “playing”). Due to the limited nature of the English verb system, previous analyses have instead focused on the productive use of verbs in languages with richer inflectional systems, such as Italian (Pizzuto and Caselli, 1992), Portuguese (Rubino and Pine, 1998), or Spanish, which permit greater levels of variability (Aguado-Orea and Pine, 2015). In English, there is only one affix to mark the third singular person in the present tense (“she wants it”), whereas in Spanish, the system is more complex, as all forms of agreement require specific suffixes. The present indicative paradigm is summarised in Table 1A.

**Table 1A:** The use of verbs with affixes in Spanish (V means verb) present tense indicative.

Agreement	Affixes	Examples	English equivalent
1. SG	V-o	(yo) salt-o	I jump
		(yo) como-o	I eat
2. SG	V-as	(tú) salt-as	you jump
	V-es	(tú) com-es	you eat
3. SG	V-a	(ella) salt-a	she jump-s
	V-e	(ella) com-e	she eat-s
1. PL	V-amos	(nosotros) salt-amos	we jump
	V-emos	(nosotros) com-emos	we eat
2. PL	V-áis	(vosotros) salt-áis	we jump
	V-éis	(vosotros) com-éis	we eat
	V-ís	(vosotros) ven-ís	we come
3. PL	V-an	(ellas) salt-an	(they) jump
	V-en	(ellas) com-en	(the) eat

Once a particular paradigm has been defined, previous analyses have attempted to determine the extent to which various grammatical operations can be deemed productive. Pizzuto and Caselli (1992) used the lowest level of analysis to examine the use of verbs by three children in longitudinal samples of Italian language. When an affix was used with two different verbs, it was deemed productive. This method enabled the observation of relatively lengthy developmental periods in which children use inflections unproductively, i.e., the period between the use of the affix with one and two verbs. This type of linguistic productivity evaluation has been applied to the analysis of other spontaneous speech data (e.g., Sultana, 2021, for the acquisition of Bangla). However, this productivity metric can be problematic for three main reasons. First, the transition between the unproductive and productive use of inflections is clearly discrete, so it is impossible to observe gradual stages. Second, the variable frequency distribution of words used in colloquial speech is also problematic, as some affixes are extremely common while others are uncommon. Thirdly, the technique is sensitive to the number of items included in the analysis, as it is simpler to identify affixes used with multiple verbs in larger samples. This is especially problematic due to the fact that longitudinal samples of speech are smaller during the early stages of development, simply because children speak more as they age. As more items are added to the analysis, it should become easier to find more combinations of verbs and affixes. However, this is not necessarily a result of a more productive knowledge, but rather a larger sample size. Tomasello and Stahl (2004) provide additional details on the combination of all three factors. They figure out how likely it is to catch at least one example of a certain feature by taking into account the variable densities of speech samples, which represent the amount of recording time per week. They find that the density needed to see the acquisition of relatively rare grammatical features increases in an exponential way.

There have been other attempts at measuring morphological productivity in samples of adult speech. One of the most salient versions is Baayen’s (2009) distinction between three ways of measuring productivity. Two of them are relevant to this study. First, realised productivity is equivalent to the idea of the size of the paradigm, illustrated above in Table 1A as six possible options for any verb stem in the Spanish Present Indicative. It is typically measured with types (unique exemplars provided by the speaker), as in Pizzuto and Caselli’s method. A second alternative consists of measuring potential productivity. It takes into consideration the number of tokens entered into the analysis (the size of the corpus). In practice, it measures the probability of a novel combination of morphemes given a sample size. Zeldes (2012) has built on this idea, developing the Productivity Complex to measure the use of constructions (not just morphemes) in a productive way. It includes not only the size of the sample and the availability of lexical items, but also the possibility of a growing vocabulary. The analyses in this article are all based on the same basic idea, but they look at changes in productive knowledge with a focus on development.

The current article systematises a relatively recent technique used to establish meaningful comparisons across 2 separate language samples (initially reported in Aguado-Orea, 2004). The technique has three primary components. First, it examines spontaneous speech samples as opposed to elicited utterances. Second, it emphasises the correct use of grammatical production as opposed to error rates. And finally, it accounts for the risks that were present in earlier analyses of productivity. First, the proportion of items used in a single form can be used as an estimate of triteness (TRI), which is defined as a lack of creativity within a system that would permit a more productive use of the grammatical features under analysis. And secondly, the average number of successful verb combinations can be used to measure creativity (CRE). CRE can adopt values ranging from one to six, as there are six possible inflections for the present tense (indicative) paradigm in Spanish. Therefore, children with low CRE scores and high TRI scores utilise the system less effectively. Critically, these methods can be used to make two separate comparisons: one between participants (typically comparing the child’s productivity to that of the parents) and one within participants (comparing two stages of development). In conclusion, the current study defines “productivity” as the observed combination of lexical items within a grammatical paradigm that could allow for such a combination.

It is important to keep in mind that the technique has been in use for nearly two decades, and that recent advancements allow for a more accurate estimation of children’s initial levels of grammatical productivity. The increased rigour made possible by the adoption of open science (Molloy, 2011; McKiernan et al., 2016) is an important factor. We have now access to well-established online data archives, such as the Open Science Framework (Tackett et al., 2019). Because other authors have access to the datasets, these archives, which did not exist two decades ago, greatly facilitate the reproducibility of these kinds of analyses. Also, open software repositories, such as GitHub (Perkel, 2016), make the actual process of analysis publicly available to the extent that the analyses can be repeated (or even modified) by other researchers. It is important to note that open science is not a recent development in the study of language acquisition. The CHILDES system, which has been in use since the 1980s (MacWhinney, 1992, 2000), is a notable illustration. It has enabled the publication of spontaneous speech datasets for a variety of languages. However, although there was a high degree of homogeneity in the transcription method, the actual application of a particular productivity criterion was not visible to the academic audience. Regardless, one of the benefits of these data repositories is the ability to conduct cross-linguistic studies, given the availability of public datasets for a variety of languages. Using regular expressions (RegEx, Friedl, 2006), it is now possible to conduct random sample extractions in a systematic manner, which is yet another factor that contributes to the increased accuracy of estimates of productive knowledge. RegEx is a computational mechanism for selecting text sequences within a corpus. It is possible to establish various criteria, such as the presence of a particular lexical item in a particular position (like the pronoun “I” preceding an auxiliary verb). It supports a variety of programming languages, including Python.

To conclude this section, the present set of analyses focuses on the productive use of subject agreement across two languages, English and Spanish, a feature that has been the focus of a substantial number of hypotheses regarding the prerequisites for early speech production. Predictions regarding the productive use of verbs with subjects are, in some respects, contradictory in both languages. The purpose of the following section is to explain why it is important to provide accurate estimates of the productive use of verbs with subjects and why identifying opposite effects is relevant to these models.

### Theoretical implications of grammatical productivity

The productive use of grammatical constructions by children (and adults) has direct implications for a theoretical debate that has impregnated not only the study of language acquisition but two central arguments in cognitive science: what type of knowledge underlies the early use of language, and how it is acquired. Two main sets of predictions have been made in the past: (1) a set of models that stem from the assumption of relatively high levels of symbolic representation (i.e., rule-based) versus, (2) a set of models defending less-organised (e.g., connectionism-based) networks. Pater (2019) provides a recent critique of this argument, and the contention between Pinker and Price (1988) and more recent revisionists, such as Kirov and Cotterell (2018), is illustrative of the significance of the theoretical conflict between the two sets of predictions.

Models based on the Principles and Parameters (P&P) approach constitute a classical example of hard constraints aiming to explain the process of learning a first grammar adopting rule-based representation (see Newmeyer, 2017, for a review of the framework). Under this approach, the core grammatical principles are shared across languages, but children must set the specific parameters for their target systems. This parameter setting mechanism is largely input-driven and simplistic in nature: when children identify an example of the value required in their language, the parameter setting process is fired. The general implication of this model is that, once the symbolic rule has been established, the system should be fully productive. Therefore, gradual stages in development are explained by means of two mechanisms: the acquisition of lexical units plus a maturational process impacting certain syntactic operations. One of the classical implementations of the P&P approach is the Structure Building Model (SBM), formulated by Radford (1990). A key feature of SBM is the gradual acquisition of lexical knowledge, in particular inflectional morphemes. Although children might set the parameters of their languages effortlessly, they still need to acquire a critical mass of lexical items to become fully productive. Thus, the piecemeal acquisition of lexical knowledge accounts for the observed levels of partial productivity. This idea is also present in other hypotheses assuming P&P with different implementations, like Hoekstra and Hyams (1998) and Valian (1991). Wexler (1998, 1999) suggests that children have knowledge about the central parameters “at the earliest observable stages, that is, at least from the time that the child enters the two-word stage around 18 months of age” (Wexler, 1998, p. 25). Opposing the SBM, Wexler believes that children have mastered lexical knowledge of the main inflections at that time too. So, the obvious question that this model must answer is how partial productivity can be explained. It proposes a maturational state, known as the Optional Infinitive (OI) Stage, working at a more cognitive-based level in the following way: Children cannot check more than one of the parameters required to express subject agreement during this period. Since some languages require a double-checking operation, it results in certain patterns of errors affecting specific sets of inflections. It explains why children exposed to overt subject languages (like English or German) fail to select the correct subject agreement in some cases (e.g., *he play), whereas children learning null subject languages (like Spanish or Italian) do not seem to make these types of agreement errors (but see Aguado-Orea and Pine, 2015), since these languages only require one parameter to check. Pinker’s (1996) paradigm-building account relies heavily on the gradual acquisition of lexical Items too, in combination with semantic constraints that gradually become available to children during a very similar maturational process. All in all, the predictions made by these models for the productive use of verbs can be summarised as follows: Once the implications of any maturational stages have been defined and considered for a given language, the only possible limitation in the productive use of grammar is the gradual acquisition of lexical items. In other words, estimates of productivity that take lexical knowledge into account should be used as important tests of these kinds of models that defend hard constraints.

The Variational Model (VM) proposed by Yang (2002) is a more recent attempt to explain gradual increases in productivity by giving relatively less importance to lexical knowledge. In this case, rather than setting parameters, the learning mechanism evaluates the competing probabilities of plausible grammars. Children detect regularities in the input and extract rules from it. This allows for a much higher level of individual variation (a problem posited for models adopting P&P), while also constituting a lighter version of innate predetermination. Therefore, the relative frequency of items included in the speech addressed to children is a contributing factor. Within his Tolerance Principle (TP), Yang (2018) formulates an equation to estimate the proportion of regular examples over the exceptions required in the input to fire a generalisation process, consequently adopting a symbolic rule. Strikingly, a key aspect of the principle is that a relatively small vocabulary supports learning, because the generalisation threshold is less restrictive (Schuler et al., 2021). But as Yang et al. (2017) acknowledge, TP is only one of the three factors comprising the design of language learning, the other two being experience and Universal Grammar (e.g., Crain, 1991), a uniform genetic architecture to interpret the environmental data as linguistically grammaticizable information. All three factors have been incorporated into a wider framework, namely the Biolinguistic Approach (Crain et al., 2017), representing a less strict version of innate predetermination. There are other models on this side of the spectrum, just to name a few, the Bayesian Grammar Acquisition model (Culbertson and Smolensky, 2012; Culbertson et al., 2013), Rational Constructivism (Xu, 2019) and the Computational Origin of Representation (Piantadosi, 2021); there is obviously no space to provide detailed descriptions of all these models here, but they all share an attempt to integrate gradual levels of productivity into systems that stem from symbolic or rule-base assumptions (e.g., Foltz et al., 2021).

On the other side of the spectrum, constructivist positions have tried to provide satisfactory explanations of incomplete productivity during the early stages of development, avoiding hard assumptions of innate prerequisites for the acquisition of grammatical knowledge (see Behrens, 2021 for a recent review). The approach originated from disciples of the Piagetian school, like Bruner and Watson (1983) and Karmiloff-Smith (1992), highlighting the limited nature of early productions by English-learning children (e.g., Braine, 1993). Early propositions included the Slot and Frame model (Pine and Lieven, 1997), building on the fact that early constructions missed the combinatorial properties predicted by defendants of hard predetermination, and the Verb Island Hypothesis (Tomasello, 1992), focusing on the gradual construction of productive knowledge around verbs. Also, in conflict with the formalist approach to linguistics (see a review in Ibbotson, 2020), constructivist models were later developed into broader postulates, like the Usage-Based Theory (Tomasello, 2000), or the Emergentist approach (MacWhinney et al., 2022). They all predict limited productive use of grammatical features during development, ruling out any hard innate predisposition to learn grammatical features. The number of current hypotheses generated under the neo-constructivist umbrella is numerous, with models giving different weights to the role of frequency (a review can be found in Ambridge et al., 2015), mechanisms of analogy and generalisation (see Ambridge, 2020, and the commentaries to the article) and the actual nature of early knowledge (Goldberg and Ferreira, 2022). These models would tend to predict that children’s productivity is dependent on the complexity of the system being acquired, in combination with the properties and regularity of the input received to acquire this system. Therefore, gradual changes would be predicted for all languages being acquired, and potential differences between the speech productivity of children and adults would depend on the complexity of the acquired grammatical paradigm.

### A new technique to estimate productive use of grammar

The richness of the theoretical production has logically been accompanied by decades of empirical research on early speech production that has fallen into two broad categories: experimental designs eliciting production from children, and the analysis of spontaneous speech. The seminal work of Berko (1958) is a paradigmatic example of the first category. She developed an ingenious method to test the ability of young children to combine novel word stems with known suffixes in English by priming them with other suffixes. For example, she would use the sentences in (1) (Berko, 1958, p. 156) to assess the present tense of the third person:

1. This is a man who knows how to naz. He is nazzing. He does it every day. Every day he ___.

An advantage of this technique is that, whenever a child manages to solve the task, we ascertain that the cognitive skills required to productively combine stems with suffixes are present. This is achieved by either generalising the example from ‘he does’, or by applying a grammatical rule (i.e., Verb+/s/). One problem, however, is the artificiality of this technique, to the extent that we cannot know if unsuccessful children already incorporate the grammatical knowledge but fail to produce the inflection because of the high demands required to solve the task, particularly with unknown words. More importantly, we do not know if a lack of production in these types of techniques indicates a partially incomplete (gradually developed) skill or a complete lack of grammatical knowledge. More recent methods have been developed in order to attenuate these problems in English (Matthews et al., 2005) and Spanish (Aguado-Orea et al., 2019) by priming constructions involving the use of verbs with subjects. In an even simpler way, researchers have directly asked children to repeat sentences that could incorporate low-frequency words (e.g., Matthews and Bannard, 2010). In all these cases, the core evidence relies on items artificially chosen by the researchers, so the actual distribution of frequency of words and sentences in the speech addressed to children cannot be estimated.

The second alternative method, the analysis of child language corpora, has represented a very important source of evidence during the last six decades of research. The collection and analysis of spontaneous speech alleviates the problem of artificiality faced by experimental designs, so it has been substantial not only in the detection of systematic patterns of errors committed by children across different languages but also in establishing the proportion of correct use across different features of language for relevant developmental stages. A salient example is the test of the hypothesised OI Stage in English and other Germanic languages. Recall that this hypothesis suggests that there is a period when children interpret the verb in a sentence like (2) (Wexler, 1994, p. 330) as a non-finite form instead of a finite one (a review can be found in Wexler, 2011).

2. *Mary play baseball

The model can thus estimate specific proportions of OI-related errors across languages. It is anticipated that error rates will be very low for Spanish and high for English and Dutch. The number of OI errors committed by children has been the primary source of evidence for evaluating these types of hypotheses (Freudenthal et al., 2015, critically evaluate the predictions with a computational model). One of the issues with estimations of incorrect usage is that these rates are predicted to be quite low for certain languages (such as Spanish and Italian). Due to the tendency for samples to be sparse during the early stages of development, incorrect uses may be insufficiently sampled or not sampled at all in the datasets, making them potentially insufficiently informative. This absence of errors is indicative of complete productivity. Analysing the extent to which verbs are productively combined with subject pronouns in correct sentences is an alternative approach to the study of subject agreement. Thus, we can draw a picture of the nature of knowledge underlying the productive use of subject agreement by children at various developmental stages and compare it to the way in which their caregivers employ these sentence structures. This is the method used by Aguado-Orea and Pine (2015) for the analysis of verb inflection in Spanish, which has also been applied to other languages with a rich inflectional system (e.g., Polish, Krajewski et al., 2011). However, even when analysing the rate of correct verb forms, sample size remains an issue when the use of certain grammatical forms is influenced by a highly skewed frequency distribution. And it is undeniable that this is the case in any sample of language, according to Zipf’s law (Yang, 2013).

Despite the fact that Aguado-Orea’s (2004) method has only been applied to languages with abundant inflection, there is no apparent reason why it would not be informative in languages such as English once the grammatical features have been clearly defined. In English, subject pronouns must agree with verbs using auxiliaries in progressive forms, for example. Therefore, a set of syntactic paradigms can be established, as well as various productivity estimates. Table 1B provides a summary of two distinct paradigms for estimating varying levels of productive use.

**Table 1B:** Possible syntactic paradigms for the analyses of productivity across Spanish and English (V means verb).

Agreement	Spanish present indicative	English progressive
1. SG	V-o	I am V-ing
2. SG	V-as	you are V-ing
	V-es	
3. SG	V-a	she is V-ing
	V-e	he is V-ing
1. PL	V-amos	we are V-ing
2. PL	V-áis	You are V-ing
	V-éis	
	V-ís	
3. PL	V-an	they are V-ing
	V-en	

As it has been mentioned above, theoretical models defending symbolic knowledge and innate predetermination would predict that children have acquired full productive use of the paradigm in both English and Spanish as soon as lexical knowledge has been achieved. The production of subject agreement has consistently been analysed by looking at the proportion of errors committed with subjects. It is still unknown if children make use of the system in a fully productive way (i.e., reaching the levels of productivity observed in adults) in languages like English, since all results have focused on error rates.

A good analysis of creativity should be able to account for the hazards described above: sample size and lexical knowledge. The number of items included in the analyses (i.e., the sample hazard, SH) must be matched across samples to provide fair comparisons of both CRE and TRI. Of course, adults are expected to produce more sentences than children. Also, children produce more sentences as they grow older, increasing the probability of finding productive constructions in later developmental stages. Although we should not disregard the fact that this larger number of combinations could be due to a more advanced level of grammatical competence, measures of productivity should be able to control for SH. Given two samples of different sizes, a way to tackle SH is to extract a series of random samples from the largest dataset, matching the size of the smaller one. The second problem is a failure to control for the lexical knowledge already achieved by children at a given point (i.e., the lexical hazard, LH). As the vocabulary of morphemes grows (also known as knowledge of the world, Yang et al., 2017), more combinations within the paradigm are possible, and again, although an increase in syntactic knowledge should not be disregarded, the increase in productivity could be explained by a larger level of lexical knowledge. In this case, the solution consists of looking only at the lexical items shared in both samples (i.e., across participants or in both developmental stages).

In sum, although the study of morphological productivity has become more prominent in recent years (Finley, 2018), particularly in studies adopting a crosslinguistic approach (e.g., Moran et al., 2018; Ambridge et al., 2021), most studies have either adopted an experimental approach, typically eliciting speech or asking children to repeat sentences, or they have looked at the errors committed by children for one specific grammatical feature and one target language, instead of looking at the production of correct sentences in spontaneous speech. Some analyses of productivity looking at richly inflected languages have been run too, but they have not been matched with other languages like English. The present study, with a strong methodological focus, aims at presenting the results of a new technique for estimating the creativity (and triteness) in the possible combinations of subject agreement in English and Spanish. The main objectives are: (1) to examine to what extent the early use of language by children is less productive than the adult one; (2) to see if children’s grammatical productivity increases developmentally for the use of subject verb agreement in English and Spanish; and, (3) to explore the potential differences in productivity observed across languages.

## Materials and methods

The present study analyses previously collected datasets to produce systematic estimations of creativity (CRE) and triteness (TRI) in Spanish and English, controlling for SH and LH.

### Design

The study is run in three stages: (1) extraction of data from the CHILDES system; (2) a systematic sampling procedure; and (3) an estimation of the potential differences in productivity.

### Participants

All datasets have been extracted from publicly available longitudinal datasets, consisting of interactions between parents and children at home. Two Spanish-speaking children from the Orea-Pine corpus (Aguado-Orea and Pine, 2015) and four English-speaking children (Annie, Eleanor, and Fraser) from the MPI-EVA Manchester corpus (Lieven et al., 2009) have been considered. Since parental speech production constitutes an important factor of the study, it also includes the speech of one of the main caregivers. Table 2 summarises the ages and genders of all 6 children.

**Table 2:** Details of participants.

Child	Gender	Target language	Start age	Final age	Total utterances
Juan	Boy	Spanish	1;10.21	2;5.29	15,945
Lucía	Girl	Spanish	2;2.25	2;7.15	10,616
Elanor	Girl	English	2;0.2	3;1.17	77,046
Fraser	Boy	English	2;0.0	3;1.11	141,992
Gina	Girl	English	3;0.1	4;7.29	226,607
Helen	Girl	English	3;0.2	5;1.19	145,755

The English corpus is larger than the Spanish one, but this should not have an effect on the estimations of productivity since all analyses are run either individually or within the adult-child direct interactions, using samples with matched sizes.

### Materials, procedure, and analyses

Two sets of constructions are extracted from the corresponding corpora and converted into lists of tokens, separated by an underscore sign, and saved as two text files (sample 1 and sample 2). For the child vs. adult comparisons, the lists of tokens are extracted from interactions between the target children and their caregivers. For the developmental analyses, the pairs of files belong to the speech of the same child in two different segments of the corpus: the total number of transcripts has been divided into two equal numbers (on two occasions, Time 1 includes one more transcript because the number was odd). All main analyses are run with the use of the Estimations of Linguistic Productivity (EsLiPro) script, written in Python for the purpose of this study, and publicly available at https://github.com/JaviAgua/EsLiPro.

**Figure 1:**
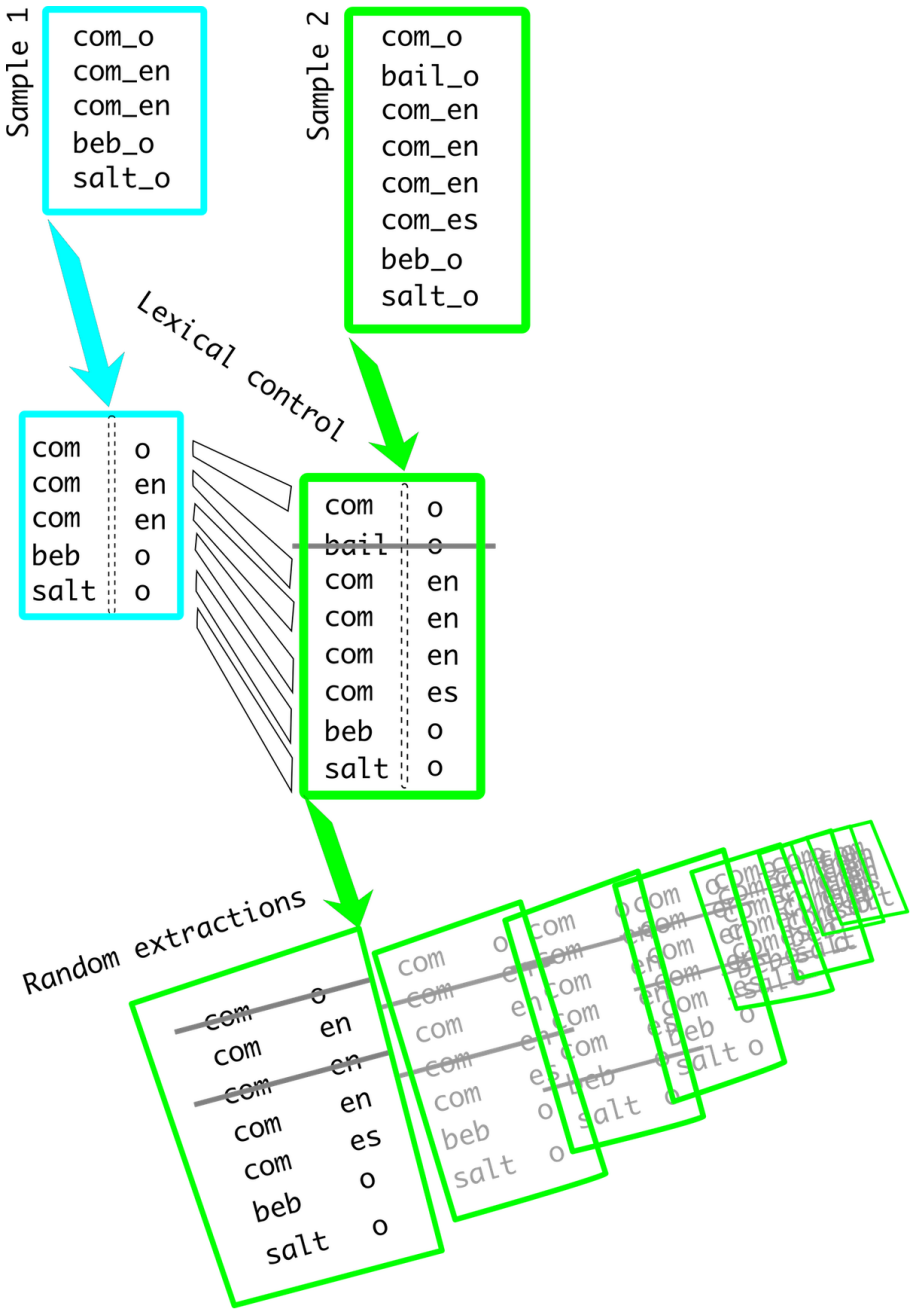
Example of the lexical and sampling controls used by EsLiPro.

The initial sets of tokens are extracted from the CHILDES datasets. The convention adopted in this system includes a line of speech followed by additional tiers with grammatical information on a word-by-word basis. For the present study, a combination of COMBO and KWAL commands has been applied to the %mor tier, where the morphological information is presented. This solves the potential problem associated with homophonous words (e.g., “help” could have been used as a noun or as a verb, and “you” could either be plural or singular), since words are not extracted, but their grammatical realisation. Recall that for Spanish, present indicative verb forms are extracted, whereas for English, present progressive is used. The following OSF repository contains all lists of CHILDES commands and resulting tokens^1^: The process of estimating both CRE and TRI is illustrated in Figure 1. Samples 1 and 2 are fed into the script. First, verbs not shared in both samples are removed from the analyses. In the example, ‘bailo’ is removed from sample 2 because no examples of that verb were found in sample 1. Then, 1,000 random sets of tokens are extracted from sample 2, matching the size of sample 1 (5 tokens in the example). EsLiPro computes the values of creativity for all prefixes and suffixes included in the corpus. For instance, CRE’s value for ‘com-‘is equal to 2 (‘-o’ and ‘-en’). The value of creativity is computed for all 1,000 sub-samples and averaged. TRI is expressed as a percentage of items used in just one possible form (not productively) out of the total number of possible combinations.

For the comparison established between the speech of children and their caregivers, the whole corpus was considered. Sample 1 consists of the child’s speech, and Sample 2 is the adult’s. For the developmental analysis, all corpora were divided into two chronological halves and treated as samples 1 and 2.

In every instance, the differences in CRE between samples 2 and 1 are computed (i.e., the result of subtracting the levels of creativity per verb). Finally, a bootstrapping analysis (Davison and Hinley, 1997) employing sampling with replacement is conducted, and the 95% confidence interval of the mean is calculated using the Bias-Correlated and Accelerated (BCa) (Efron, 1987) and the percentile methods, as both are conservative enough (Henderson, 2005). The analysis is run with the ‘boot’ package (version, 1.3–28, Canty and Ripley, 2021) using the R Statistical Language (version 4.1.2, R Core Team, 2021). Since the confidence intervals for both methods (BCa and percentile) were nearly identical, only BCa is reported here for the sake of simplicity. The script and outputs are available at https://osf.io/8s2w3/.

## Results

This section is organised around two main groups of analyses of productivity: (1) a comparison of the values of CRE and TRI observed for subject agreement in children and adults; and (2) the equivalent sets of analyses for developmental changes.

### Comparisons of productivity between children and their caregivers

The most productive verbs in the speech of Juan are ‘hacer’ [= to do] (5 inflections), ‘querer’ [= to want], or ‘caer’ [= to fall] (both with 4 inflections). The productivity observed for the corresponding parental speech, averaged for 1,000 random sample extractions, also includes relatively high numbers for these verbs: 4.81 inflections for ‘hacer’, 3.48 for ‘querer’, and 2.20 for ‘caer’. Other verbs were more productively used by the adult, like ‘poner’ [= to put] (2 inflections in the child’s speech, 4.38 in the adult one), and other verbs were more productively used by the child, like ‘mirar’ [= to look], used with two inflections by Juan and only one by Juan’s father.

The observed evidence is 13ioarised in Table 3. The first rows include the values of productive use for Juan and Juan’s father, and the following rows include the respective values for Lucía and Lucía’s father.

**Table 3:** Descriptive results for the productivity observed in the speech of Spanish speaking children and their respective caregivers.

Control	Sample	CRE (infl per verb)	sd	Tokens	Verbs	TRI%
None	Juan	1.83	1.14	3,045	145	53.79
None	Juan’s father	2.14	1.43	8,308	268	48.13
LH	Juan	1.86	1.15	3,040	140	52.14
LH	Juan’s father	2.74	1.55	7,928	140	26.43
LH and SH	Juan’s father	2.19	1.34	3,045	140	26.43
None	Lucía	1.61	0.93	1,609	74	63.51
None	Lucía’s father	2.13	1.40	4,254	172	49.42
LH	Lucía	1.64	0.95	1,605	70	61.43
LH	Lucía’s father	2.89	1.41	3,859	70	24.29
LH and SH	Lucía’s father	2.35	1.24	1,609	70	24.29

Table 3 shows how controlling for lexical knowledge increases the level of productivity in all samples. This is because most of the verbs used in only one form tend to be rare in colloquial speech, and hence they were used by only one of the participants. Controlling for both lexical knowledge and sample size also has a positive effect on the level of CRE over a lack of any controls, but the impact is smaller in this case.

Regarding the levels of TRI, the effect caused by the control of lexical knowledge is much higher in the speech of both adults. Juan’s father produced 48.1% of verbs with just one inflection in the whole sample. When the verbs not produced by Juan are removed from the analysis, the percentage is 26.43% (the extraction of random samples has no impact on the values of TRI). The effect is similar in the case of Lucía’s caregiver; an initial value of 49.42% gets down to 24.29%.

**Figure 2:**
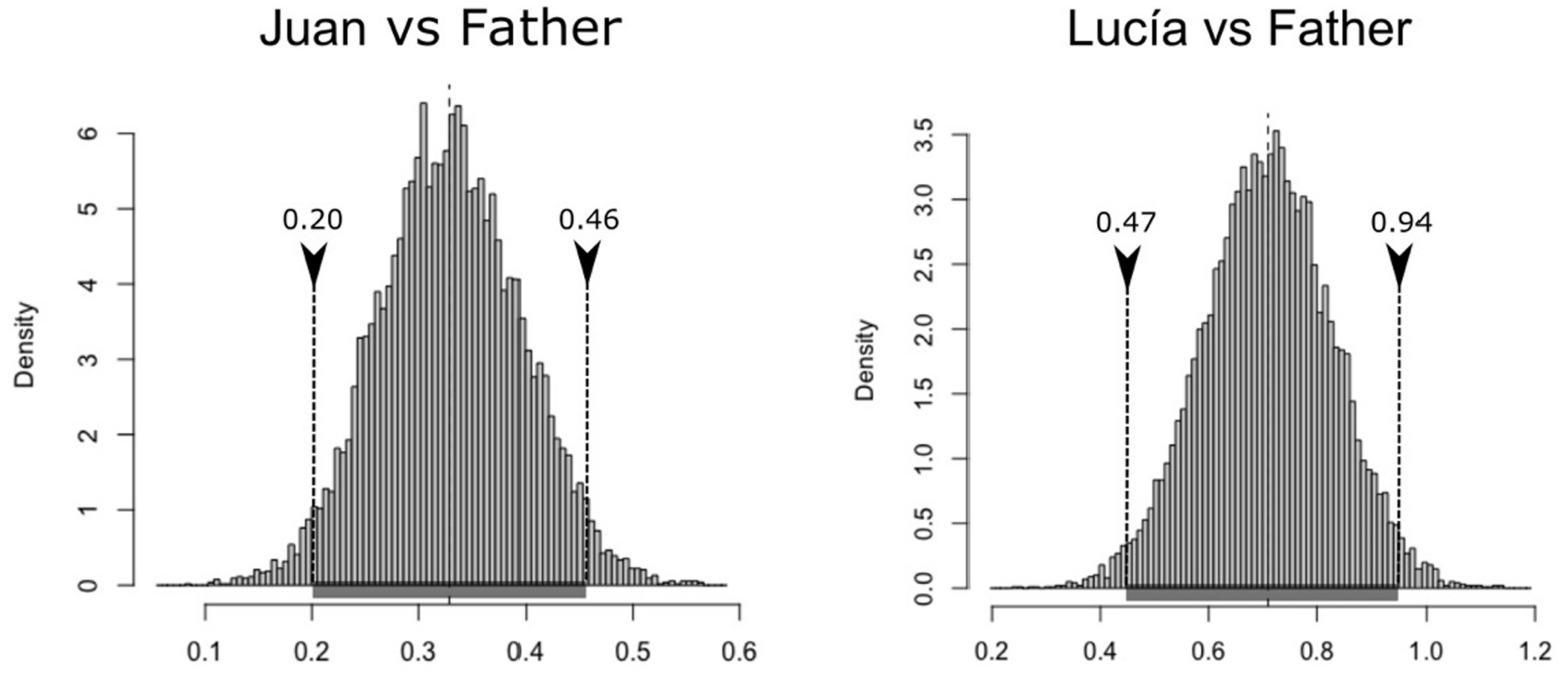
Histogram of the bootstrapped replicates of the differences in inflections per verb between the parental and child speech in Spanish.

To respond to the question about the potential differences in creativity, an ordinary non-parametric bootstrap method has been used, to avoid making *a priori* inferences about the distribution of these differences. First, all differences in the number of inflections used per verb were calculated across both samples of participants in the following way: CRE values observed in the parental speech minus CRE values observed in the child’s speech, on a verb-by-verb basis (M_JuanvsAdult_ = 0.33, sd = 0.79; M_LucíavsAdult_ = 0.71, sd = 1.00). Then 10,000 random samples with replacement have been extracted with a confidence interval at the 95% level that does not incorporate the null value (μ_Bca,low_ = 0.20; μ_Bca,high_ = 0.46) (Figure 2, left) in the difference in productivity between Juan’s father and Juan, nor in the difference between the values observed for Lucía’s father and Lucía (Figure 2, right) (confidence interval: μ_Bca,low_ = 0.47; μ_Bca,high_ = 0.94). Therefore, more creativity is predicted in parental speech after controlling for sample size and the vocabulary of verbs.

The pattern of results observed for the use of present progressive forms in English is substantially different from the one observed for present indicative in Spanish. The differences in the productive use of verbs with different subject pronouns are less dissimilar between adults and children, as shown in Table 4. The levels of creativity observed for children are very similar to the ones observed for adults. The value of CRE for Fraser’s mother increases when the lexical control is introduced, and the amount of TRI is sensibly reduced too.

**Table 4:** Descriptive results of the productivity observed in the speech of English-speaking children and their respective caregivers.

Control	Sample	CRE	sd	Tokens	Verbs	TRI%
None	Eleanor	1.89	1.33	1,657	123	56.10
None	Eleanor’s mother	1.83	1.33	5,962	248	59.68
LH	Eleanor	2.01	1.35	1,630	101	49.50
LH	Eleanor’s mother	2.65	1.65	4,846	101	31.68
LH and SH	Eleanor’s mother	1.96	1.22	1,657	101	31.68
None	Fraser	2.30	1.49	2,366	166	42.17
None	Fraser’s mother	2.38	1.65	8,889	277	46.93
LH	Fraser	2.44	1.52	2,339	147	37.41
LH	Fraser’s mother	3.35	1.67	8,560	147	19.05
LH and SH	Fraser’s mother	2.32	1.35	2,366	147	19.05
None	Gina	1.87	1.33	1828	180	57.78
None	Gina’s mother	1.96	1.34	3,023	228	55.26
LH	Gina	2.13	1.43	1760	134	46.27
LH	Gina’s mother	2.33	1.50	2,816	134	42.54
LH and SH	Gina’s mother	2.03	1.32	1828	134	42.54
None	Helen	2.29	1.57	3,480	217	46.54
None	Helen’s mother	2.25	1.57	5,594	263	47.15
LH	Helen	2.54	1.62	3,413	176	38.07
LH	Helen’s mother	2.74	1.68	5,422	176	30.68
LH and SH	Helen’s mother	2.39	1.54	3,480	176	30.68

For instance, Fraser used the verb ‘play’ with all six subject pronouns, compared to the 4.88 pronouns used by Fraser’s mother (averaged across 1,000 random samples). For other verbs like ‘stand’, the adult was more productive, with 4.55 inflections, compared to the 3 inflections used by the girl for this verb.

**Figure 3:**
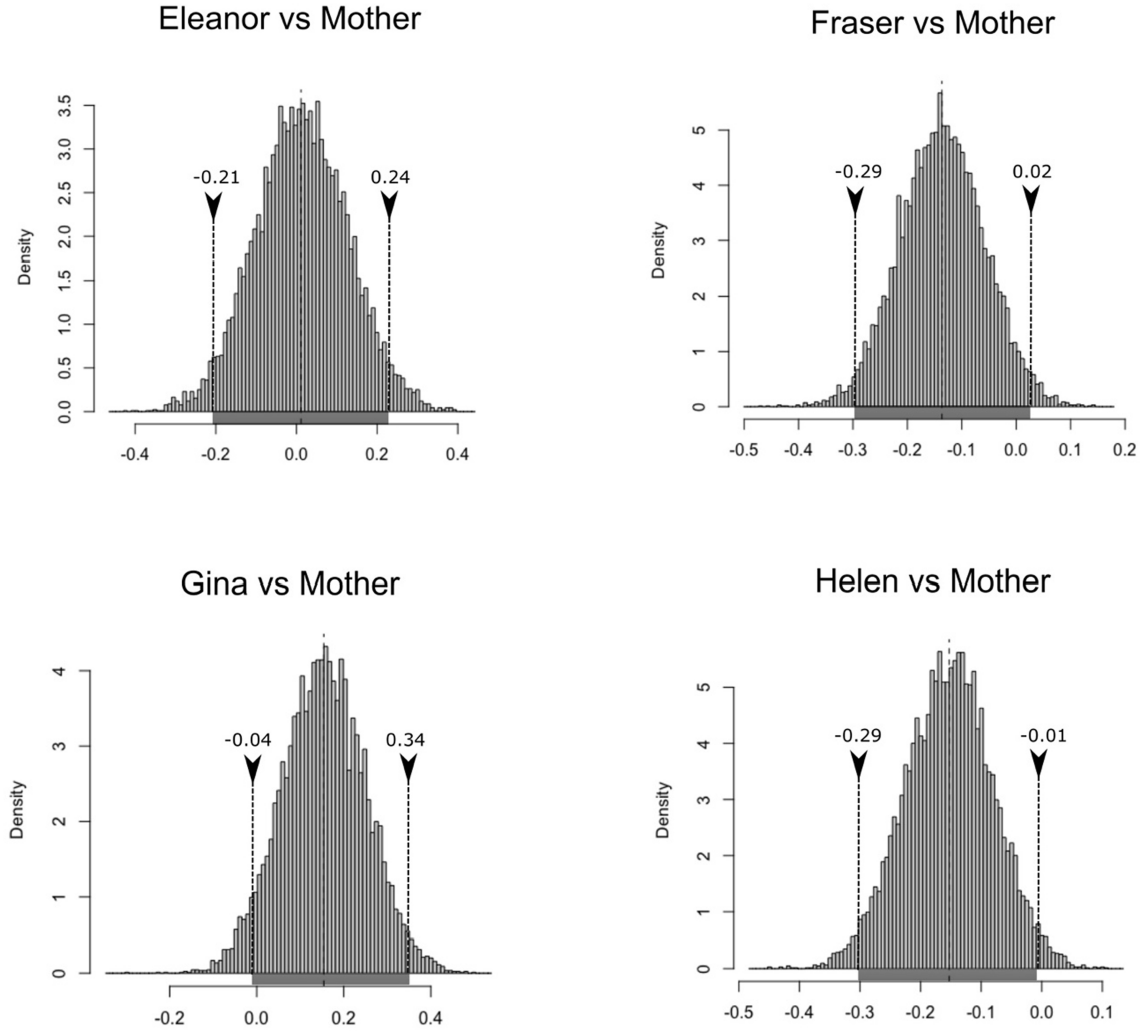
Histogram of the bootstrapped replicates of the differences in inflections per verb between the parental and child speech in English.

After running the bootstrap analyses with the list of differences observed per verb (adult minus child) in three cases, the 95% highest density interval of confidence for the mean incorporates the null value in the comparisons established between the child and parental level of creativity (Elanor: μ_BCa,low_ = −0.21; μ_BCa,high_ = 0.24||Fraser: μ_BCa,low_ = −0.29; μ_BCa,high_ = 0.02|| Gina: μ_BCa,low_ = −0.04; μ_BCa,high_ = 0.34|| Helen: μ_BCa,low_ = −0.30; μ_BCa,high_ = −0.01). Figure 3 illustrates this situation, with the high and low values of the interval indicated in the lower part of the distribution figures (grey line). This has obvious implications for the underlying knowledge of subject agreement across both languages because it indicates that it is different in nature. Although technically the difference observed in the values of creativity between Helen and Helen’s mother does not incorporate the null value, there would be a small negative trend (meaning that, after all the controls have been applied, the girl used more verbs per subject pronoun).

The mean differences for the figures are sufficiently illustrative of the similarity in the levels of creativity observed for children and parents (the whole analysis can be accessed at https://osf.io/8s2w3/).

### Developmental analyses

The results presented in this second section are a parallel version of the ones provided above, but in this case, only the data collected for children is entered into the analyses. In the case of the two Spanish children, results show a tendency to use verbs in a more productive way in the relatively short period of time considered in the analyses. The first segment incorporates the speech of Juan between the ages of 1;10.21 and 2;2.16, and the second segment corresponds to transcripts collected between the ages of 2;2.22 and 2;5.29. For Lucía, the segments correspond to the following periods: 2;2.25 to 2;4.20, and 2;4.24 to 2;7.14. Therefore, in the limited time of a few more than 3 months, children use verbs with more subject-agreeing inflections. Table 5 summarises the levels of creativity in both children’s speech over time.

**Table 5:** Developmental analysis for the Spanish children.

Control	Sample	CRE (infl per verb)	sd	Tokens	Verbs	TRI%
None	Juan Time 1	1.42	0.82	782	84	73.81
None	Juan Time 2	1.83	1.17	2,264	123	56.10
LH	Juan Time 1	1.52	0.90	748	62	67.74
LH	Juan Time 2	2.26	1.32	2087	62	40.32
LH and SH	Juan Time 2	1.79	1.01	782	62	40.32
None	Lucía Time 1	1.56	0.80	929	48	60.42
None	Lucía Time 2	1.42	0.71	680	57	68.42
LH	Lucía Time 1	1.84	0.86	897	31	41.94
LH	Lucía Time 2	1.61	0.80	632	31	54.84
LH and SH	Lucía Time 2	1.67	0.76	929	31	41.94

**Figure 4:**
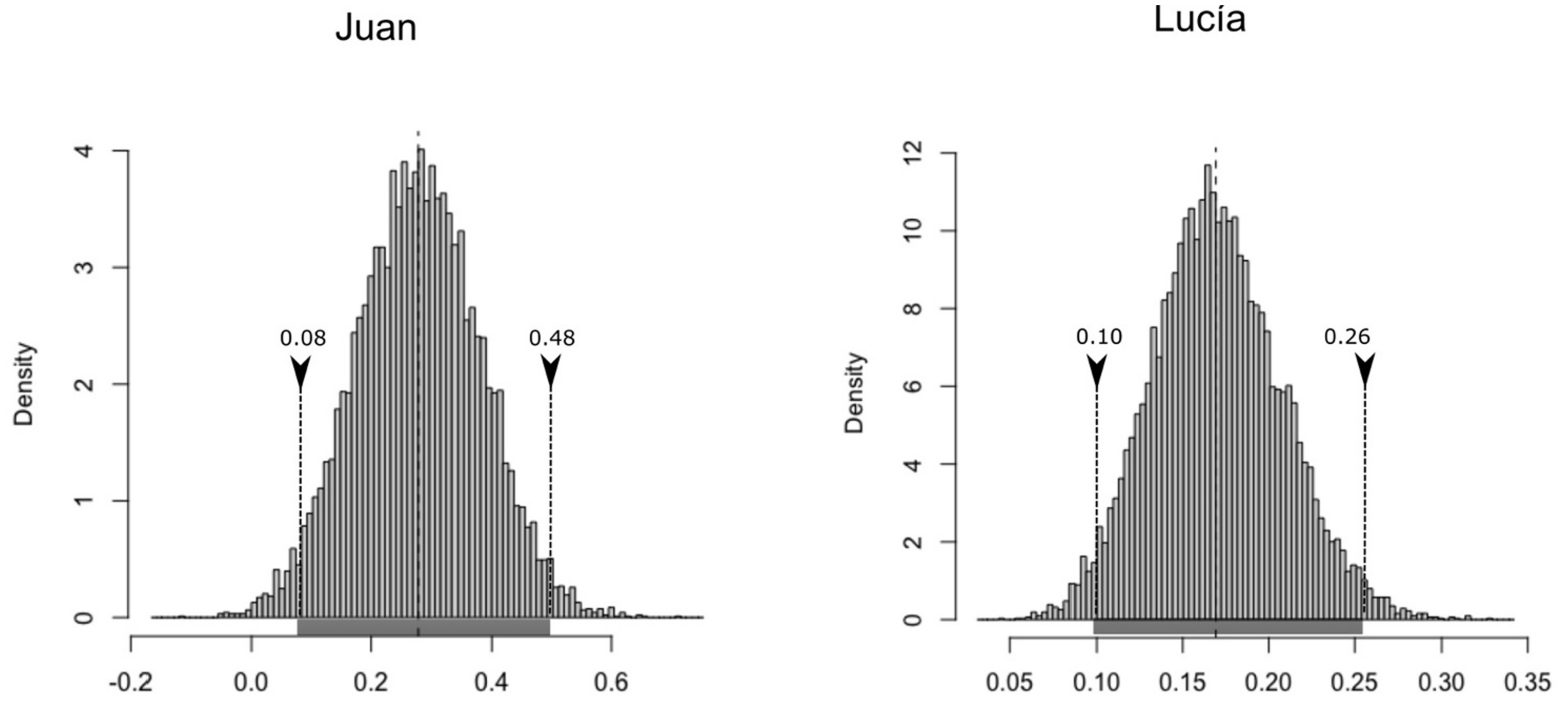
Histogram of the bootstrapped replicates of the differences in inflections per verb at Time 2 and Time 1 in the two Spanish speaking children.

The introduction of lexical control increases the level of productivity in both developmental points. It can be observed in the values of creativity and in the decrease of TRI. Another bootstrapping procedure was used to test the hypothesis that verbs were more productive in stage two (compared to stage one). The 95% highest density interval of confidence (μ_BCa,low_ = 0.08; μ_BCa,high_ = 0.48) does not incorporate the null value in the differences of levels of creativity observed in the two developmental samples of Juan’s speech (Figure 4, left). The result is very similar to that in the analysis of Lucía’s production (Figure 4, right), with the following confidence interval: μ_BCa,low_ = 0.10; μ_BCa,high_ = 0.26. In sum, it is likely to be expected that the creative use of verbs will increase between Time 1 and Time 2 after controlling for sample size and vocabulary of verbs.

It has been found that children and parents use verbs with subjects at equivalent levels of productivity in English. The corresponding developmental analysis also shows this pattern (lack of an increase in productivity). Results for the English children are summarised in Table 6.

**Table 6:** Developmental results for the English speaking children.

Control	Sample	CRE	sd	Tokens	Verbs	TRI%
None	Eleanor Time 1	1.72	1.16	649	74	59.46
None	Eleanor Time 2	1.81	1.19	1,008	93	58.06
LH	Eleanor Time 1	2.00	1.29	608	44	45.45
LH	Eleanor Time 2	2.55	1.35	938	44	27.27
LH and SH	Eleanor Time 2	2.18	1.15	649	44	27.27
None	Fraser Time 1	1.95	1.28	990	115	51.30
None	Fraser Time 2	2.14	1.42	1,376	132	48.48
LH	Fraser Time 1	2.22	1.39	916	81	40.74
LH	Fraser Time 2	2.67	1.56	1,270	81	34.57
LH and SH	Fraser Time 2	2.37	1.40	990	81	34.57
None	Gina Time 1	1.71	1.16	951	119	63.03
None	Gina Time 2	1.66	1.15	877	137	63.50
LH	Gina Time 1	2.04	1.31	895	77	48.05
LH	Gina Time 2	2.09	1.37	782	77	42.86
LH and SH	Gina Time 2	1.95	1.25	951	77	48.05
None	Helen Time 1	2.12	1.50	2072	165	53.33
None	Helen Time 2	1.97	1.40	1,279	154	57.14
LH	Helen Time 1	2.54	1.60	1929	104	38.46
LH	Helen Time 2	2.32	1.55	1,199	104	45.19
LH and SH	Helen Time 2	2.21	1.44	2072	104	38.46

**Figure 5:**
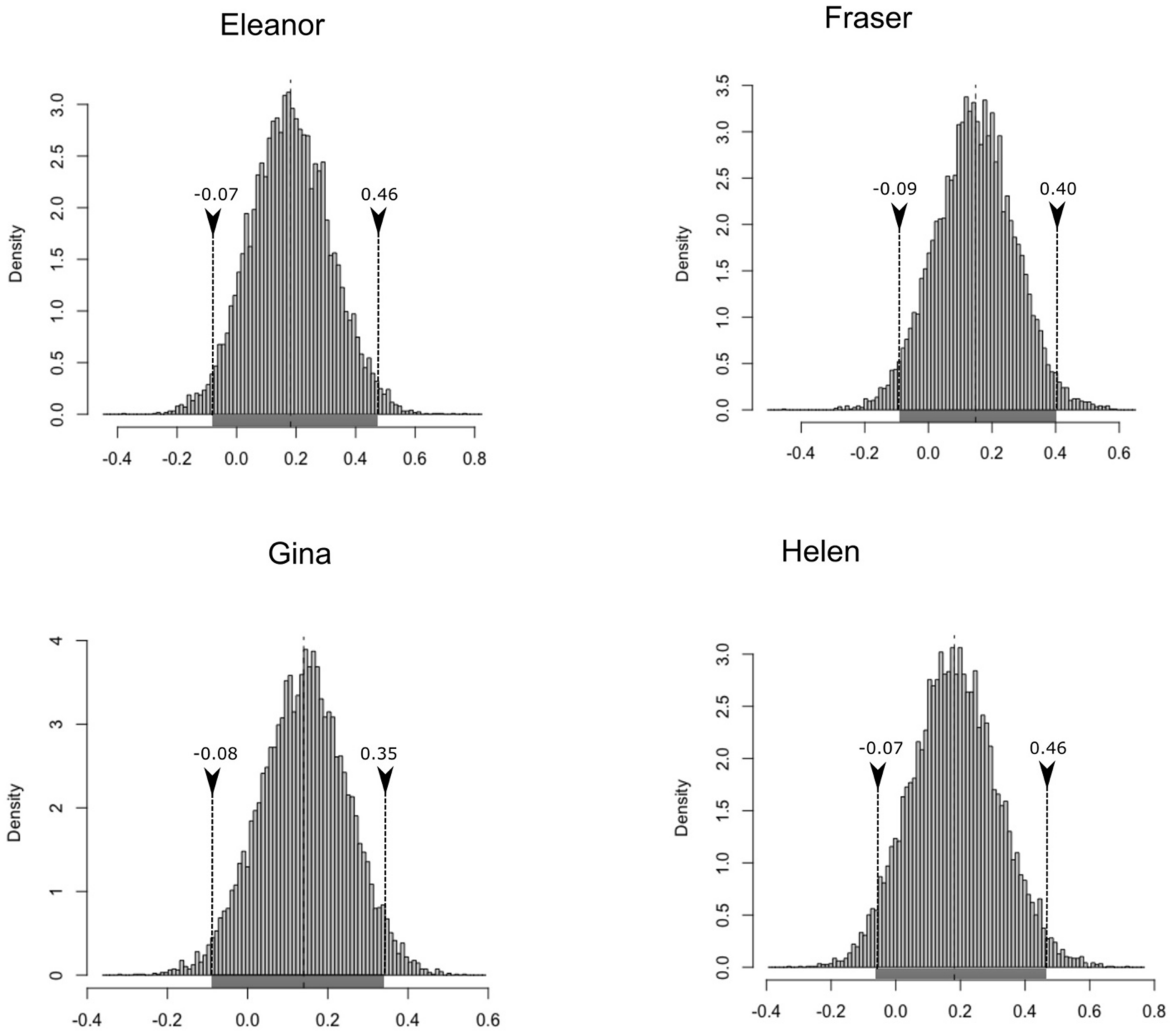
Histogram of the bootstrapped replicates of the differences in inflections per verb at Time 2 and Time 1 in the four English speaking children.

Elanor and Fraser did use verbs in a slightly more productive way in Time 2, but this was not observed for Gina and Helen. It should be remembered that the data collected for these children started later in development than the data collected for Eleanor and Fraser. The bootstrap analysis does include the null value in all four cases (Elanor: μ_BCa,low_ = −0.07; μ_BCa,high_ = 0.46||Fraser: μ_BCa,low_ = −0.09; μ_BCa,high_ = 0.40||Gina: μ_BCa,low_ = −0.08; μ_BCa,high_ = 0.35|| Helen: μ_BCa,low_ = −0.07; μ_BCa,high_ = 0.46), as illustrated in Figure 5, indicating that finding developmental changes in productivity is extremely unlikely for this paradigm.

In sum, there is an opposite pattern in the creative use of verbs with subject-agreeing markers. The creative use of verbs with different inflections gradually increases in the speech collected for Spanish-speaking children. Alternatively, the creative use of verbs with auxiliaries and subject pronouns seems to be very productive from very early in development. In the following section, the possible reasons for this patterning variation across languages are explored.

## Discussion

Capturing developmental mechanisms is a challenging task (Benton, 2022). The present study systematises a new technique to examine changes in productivity at two levels: across participants, comparing the early use of language by children against their caregivers, and within participants, trying to detect developmental changes in the productive use of grammatical features. The use of subject verb agreement has been analysed cross-linguistically (in English and Spanish). One of the advantages of looking at the productive use of verbs in correct contexts is that different estimations of linguistic productivity can be established, and hence a set of gradual points towards mastering the final system can be drawn. Of course, analyses of errors are also highly informative, and this study has no intention of providing a supplementary method, but a complementary one. In this sense, it is interesting to observe that, precisely for English, it probably makes a lot of sense to look at the proportion of correct provision because, in fact, children seem to be as productive as their parents in the use of perhaps the most frequent way to express the present tense in English, the progressive form. Thus, it should not come as a surprise that most analyses of early grammatical knowledge have focused on error rates, since researchers must have intuitively assumed that the correct use of subject agreement was uninteresting. The present study has shown that the situation is different when the correct production of the most frequent paradigm to express the present tense in Spanish is analysed. Similar findings have already been reported in Aguado-Orea and Pine (2015), but the present study offers a systematic way to approximate the levels of linguistic productivity observed in correct sentences. It expands these findings by adding a developmental analysis and a crosslinguistic comparison. The observed effects are opposite across both languages, but it is important to bear in mind that they consist only of two children acquiring Spanish and four children acquiring English. Therefore, although the present results do not show a significant difference between the productive use of subject pronouns by children and caregivers, more analyses are required before being able to conclude that a discrete difference is observed across languages. It is also important to keep in mind that the longitudinal samples comprise a later developmental stage (at least in terms of chronological age) for the four English children, so future analyses of younger children for this language may be more informative.

At a theoretical level, the results reported here have significance for our understanding of the nature of early cognitive systems, allowing us to productively use verbs with subjects in sentences. The introduction to this study has described the implications of finding effects that match better with symbolic rule-based production (like the one observed for the present progressive in English here), against those that seem to be less organised or highly sensitive to the distributional properties of colloquial speech. One of the most important implications of the early use of language in an apparently fully grammatical way is that innate constraints are supported. This was the argument defended by models adopting the P&P approach. The explicit assumption made by Wexler (1998) is that children would be fully productive once they have acquired the lexical knowledge of inflection, and this would happen very early in development. Wexler believes that in languages like English, German, or Dutch, children go through an Optional Infinitive (OI) stage, when they would be failing to check two parameters. His prediction is interpreted in proportions of errors, consisting of non-finite forms in finite contexts. Alternatively, they would not commit errors in languages like Spanish or Italian, because they would not go through this OI stage. The expected results when looking at the productive use of subject agreement in these two languages in correct sentences should reflect this maturational effect in English (with reduced levels of productivity) and Spanish. Ironically perhaps, the results presented here show that, when we look at the correct use of grammar, the opposite pattern is found: children acquiring Spanish are not fully productive, whereas children acquiring English seem to be so. These results are no less problematic for the paradigm building account (Pinker, 1996), as predictions are less clearly limited to errors and more directly linked to productive use in correct contexts. Furthermore, it is difficult to explain, according to this model, why English-learning children are so different from Spanish-learning children. Adopting a more experimental approach, Culbertson and Smolensky (2012) have formulated a Bayesian Grammar Acquisition (BGA) model to explain the increasing levels of productivity. BGA is a model based on artificial grammar learning (e.g., Reber, 1967), aimed at explaining the combination of nouns and adjectives, and nouns and numerals. It works in two stages: a set of initial biases are implemented into priors (representing the ‘innate’ component of the system), and the probability of assigning the correct hypothesis given a set of data (input) as posteriors. The initial biases are the assumed sensitivity to the difference between substantive, adjective, and numeral categories, whereas the core mechanism (the posterior) is a probabilistic context-free grammar learning process. As a result, statistical learning is a required component for shaping the core work of innate-driven knowledge (Culbertson et al., 2013). Even though this is a good example of an attempt to solve the tension between empiricist and rationalist explanations within a single model, Dupre (2021) has criticised it for being based on a very limited set of constructions: combinations of nouns and adjectives. Alternatively, the present study presents a more powerful set of paradigms, present indicative in Spanish, present progressive in English, and it shows that statistical learning is not always visible in the system (as in English, here). The opposite view to symbolic rule-based assumptions is Ambridge’s (2020) Radical Exemplar model, since it assumes that this type of symbolic knowledge might not even be reached at an adult level (Bod, 2006), but the current results show that, at least for the use of the paradigm considered here in English, there is a highly productive system that does not seem to be subjected to the sensitivity of exemplars in the early use of present progressive forms. Of course, it could be assumed that children have strong analogical powers from very early on, allowing for rule-like use, so this model (and other similar ones) must be able to explain why this analogical power is not equally effective in the Spanish paradigm considered here. In any case, the results presented in this study open the door for further analyses and challenges for current models trying to explain early knowledge from either the Constructivist Spectrum or the Biolinguistic Approach. New analyses generated with this methodology could be looking at progressive forms in Spanish too, although this type of construction is probably less common than the present indicative. Another possible set of analyses could look at other developmental stages, particularly for the analysis of English speech. The present study does not consider other factors that future studies could clarify, like the potential role of irregular forms, which are typically highly frequent, and the transparency of the paradigm, since Spanish affixes are unstressed while English overt pronouns are stressed.

This is why one of the most important objectives of the present study is fundamentally methodological. The software developed for the analyses of productivity included here (EsLiPro) is open source, so any researcher can apply it to any feature regardless of the system and language under analysis. Almost by definition, any syntactic property can be expressed as a combination of two sets of items (e.g., verb plus inflection or subject plus verb), and therefore, any corpus can be summarised into lists of tokens expressed as a combination of lexical items (e.g., stem-suffix). Once this condition is satisfied, EsLiPro can be fed with two different sets of tokens, and the vocabulary of stems and affixes is checked automatically, followed by a series of random sample extractions from the largest list of tokens, matching the size of the smallest one. The main goal of this article, then, is to give researchers who are interested in how people learn languages a simple tool that lets them do controlled analyses of grammatical productivity.

## Data availability statement

Publicly available datasets were analyzed in this study. This data can be found at: https://osf.io/8s2w3/.

## Ethics statement

This study analyses publicly available data, collected in previous studies. Ethical review and approval was not required for the study on human participants in accordance with the local legislation and institutional requirements. Written informed consent to participate in this study was provided by the participants’ legal guardian/next of kin.

## Author contributions

The author confirms being the sole contributor of this work and has approved it for publication.

## Conflict of interest

The author declares that the research was conducted in the absence of any commercial or financial relationships that could be construed as a potential conflict of interest.

## Publisher’s note

All claims expressed in this article are solely those of the authors and do not necessarily represent those of their affiliated organizations, or those of the publisher, the editors and the reviewers. Any product that may be evaluated in this article, or claim that may be made by its manufacturer, is not guaranteed or endorsed by the publisher.

## Footnotes

^1^ https://osf.io/8s2w3/
